# Something Old and New in the Treatment of Acute Dysentery

**Published:** 1887-01

**Authors:** A. B. Fordyce

**Affiliations:** Union Springs, N. Y.


### *SOMETHING OLD AND NEW IN THE TREATMENT OF ACUTE DYSENTERY.*

BY B. A. FORDYCE, M. D., Union Springs, N. Y.

I can better illustrate this announcement by reporting the particulars of a case typical of the class treated by the method claimed to be new.

It will be remembered that July and August of the past summer were noted for extremely hot days and cold, chilly nights, with heavy dews. Precisely the weather and condition of atmosphere, credited by all observers and writers upon this disease, with being the principal cause of the sporadic and often of the epidemic form of the disease. Many cases occurring at this time, in different parts of the State, were, without doubt, produced by these causes.

The disease was not epidemic in the locality of this patient, but quite a number of people in the vicinity came under observation and treatment, who had been exposed continuously to the extreme heat of the day and cold of the evening, without suitable change of clothing for change of temperature.

The case to which I refer, to show the treatment, was, in every particular, well enough marked to identify it as a violently acute form of dysentery. The patient was over sixty years of age, engaged daily, and often late in the evening, in active business during these months until the time of the attack. For two days prior to the appearance of well-marked diagnostic symptoms of this disease, he complained of languor with a sense of distension or fulness of the abdomen, not dependent upon any recognized irregularity of the digestive organs, which caused a degree of discomfort sufficient to develop uneasiness and apprehension. On the afternoon of August 26th, after a hard day's work, the first pathognomonic symptoms were noticed: A sense of coldness (not a chill), nausea and frequent desire to evacuate the bowels, attended with severe tenesmus, and without the relief usually experienced. The dejections were fluid and small in quantity, consisting from the first of mucus, mixed with blood. The urgent demand returned every fifteen or twenty minutes, and was nearly uncontrollable, while the cold sensation gradually increased, accompanied with painful dysuria. In about three hours the nausea resulted in free emesis, but without relief. Hot applications to the extremities and mustard over the stomach were applied, and morphine administered, but quickly rejected, by the stomach. Then morphine suppositories were tried, but were not retained for sufficient time to give any relief. On the next day, after passing a sleepless night, one ounce of Epsom salts, dissolved in a half pint of water, was given and retained in the stomach for sufficient time to act as a cathartic. Only small movements were produced, with but temporary relief. The same sense of fulness returned, and continued with tormina and urgent desire to have something removed.

On the following day, after another night of excruciating agony, the continuance of the apparent fulness of the alimentary canal, above the diseased part, induced me to use another cathartic—this time of castor oil, two ounces; turpentine, two drams, mixed—which was retained by the stomach, and acted freely, greatly relieving the distension and pain. I now felt quite confident of permanent relief, only to be disappointed by a return of all the distressing symptoms eight hours after. The same sensation of some solid substance in the rectum that must be gotten rid of, again became the prominent symptom. My interest in the patient had also increased, and I made a digital examination of the rectum. I found a small free space above the sphincter that would hold about half an ounce. Into this *cul de sac* the fluids in the bowel from above accumulated, and as soon as the space was filled, the tenesmus commenced, and an immediate evacuation followed. Above this space the whole calibre of the intestine was occluded with soft, pulpy, œdematous, mucus tissue, which was nearly impervious.

This condition is well described by nearly all our modern authors, viz.: Neimeyer, Bennett, Watson, Flint, Pepper in "System of Medicine," and Woodward in the second volume of "The Surgical and Medical History of the War of the Rebellion." In this last work are the reports of army surgeons, detailing the results and post-mortem examinations of nearly one thousand cases.

The inflamed œdematous condition of the mucus membrane, and consequent obstruction to passages from the bowel, being fully proved, the grave question arose, What are you going to do about it? I remembered the relief often obtained from hot water applied to inflamed surfaces, and the beneficial effects of solutions of bichloride of mercury upon ulcerated surfaces, particularly of mucus membrane, and the thought occurred to me, that if I could carry the hot water through the stricture, and above it into the bowel, allowing it to flow back in sufficient quantity to remove for a distance from above all fœcal and offending matter, I should accomplish two important objects, *i. e.*, relief of pain, and prevent absorption of poisonous matter into the blood, the antiseptic properties of the bichloride aiding in this latter object. Upon this I acted, using a soft rubber tube attached to a Davidson's syringe, passed carefully through this sensitive inflamed tissue, so as to carry the liquid above the rectum into the colon. The patient was placed on his side, with an oilcloth beneath him, and four or five quarts of water as hot as could be borne were injected and allowed to flow back with whatever substance had accumulated or remained in the bowel above. When the water returned clear, then a quart or more of the solution of bichloride, about 1 to 10,000, was injected and allowed to return in the same manner. The effect was immediate relief of pain and tenesmus. A suppository of opium, one grain, was given and retained, and, for the first time after the attack, the patient slept seven hours, awaked refreshed, could take some food, and, if perfectly still, was free from pain. In about twelve hours slight return of pain was felt, and the same treatment repeated. This treatment was continued, with the bichloride solution, four times, and the hot water alone was used for four or five days more, with suppositories of one-half grain opium, morning and night, with perfect recovery, no medicine being administered by the stomach, except one-grain doses of quinine, three times per day.

In six days all the functions of the alimentary canal were restored.

The loss of flesh in this patient, in twelve days, was twenty pounds.

This treatment was used in four other cases, but earlier in the disease, and all were promptly relieved.

It is well known that the cicatrices, from healed ulcers of the rectum, caused by dysentery, often remain to modify the form of fœcal discharges for a year or more after recovery. Several writers also mention the fact that stricture of the bowel, and cancer are produced by these cicatrices. But in all these cases the perfectly cylindrical, smooth form was acquired as soon as convalescence was established.

I have failed to find this plan of treatment mentioned by any authority. I have not tried it in chronic dysentery, but I think it is entitled to favorable consideration when it is known that so many of this class of patients, after all established remedies have been tried, are turned over to the undertaker. This, too, taken in connection with the fact that cicatrices, produced by the action of bichloride of mercury, are soft and flexible, commends any plan of treatment that promises more satisfactory results. I should not hesitate to use it in the chronic form of the disease, where ulceration was known to exist at any time prior to perforation of the intestine.

